# Extracellular Metalloproteinases in the Plasticity of Excitatory and Inhibitory Synapses

**DOI:** 10.3390/cells10082055

**Published:** 2021-08-11

**Authors:** Grzegorz Wiera, Jerzy W. Mozrzymas

**Affiliations:** Department of Biophysics and Neuroscience, Wroclaw Medical University, 50-368 Wroclaw, Poland

**Keywords:** metalloproteinase, proteolysis, synaptic plasticity, GABA, inhibitory synapse, LTP, adhesion, perineuronal nets, MMP, learning

## Abstract

Long-term synaptic plasticity is shaped by the controlled reorganization of the synaptic proteome. A key component of this process is local proteolysis performed by the family of extracellular matrix metalloproteinases (MMPs). In recent years, considerable progress was achieved in identifying extracellular proteases involved in neuroplasticity phenomena and their protein substrates. Perisynaptic metalloproteinases regulate plastic changes at synapses through the processing of extracellular and membrane proteins. MMP9 was found to play a crucial role in excitatory synapses by controlling the NMDA-dependent LTP component. In addition, MMP3 regulates the L-type calcium channel-dependent form of LTP as well as the plasticity of neuronal excitability. Both MMP9 and MMP3 were implicated in memory and learning. Moreover, altered expression or mutations of different MMPs are associated with learning deficits and psychiatric disorders, including schizophrenia, addiction, or stress response. Contrary to excitatory drive, the investigation into the role of extracellular proteolysis in inhibitory synapses is only just beginning. Herein, we review the principal mechanisms of MMP involvement in the plasticity of excitatory transmission and the recently discovered role of proteolysis in inhibitory synapses. We discuss how different matrix metalloproteinases shape dynamics and turnover of synaptic adhesome and signal transduction pathways in neurons. Finally, we discuss future challenges in exploring synapse- and plasticity-specific functions of different metalloproteinases.

## 1. Introduction

Neuroplasticity is often defined as the ability of neural networks in the brain to change through various growth, reorganization, or other modulatory processes to adapt to an organism’s variable environment and change with experience. Several aspects of neuroplasticity were revealed, including structural changes at various scales, regulation of excitability, but the most extensively studied is the ability to alter neuronal connectivity in response to different activity patterns; the phenomenon referred to as synaptic plasticity. Synapses connecting distinct neurons are greatly diversified, and any specific synapse might experience several types of plasticity mechanisms (expressed pre- or postsynaptically), depending on the stimuli eliciting it. Plastic changes at a synapse may weaken or strengthen the connection and rely on a myriad of signaling pathways and molecular players. The last decade or so proved particularly fruitful in bringing convincing evidence that synaptic plasticity and changes in neuronal excitability provide an important substrate for the processes of learning and memory storage [1,2,3]. A significant breakthrough in studying the mechanisms of synaptic plasticity was the discovery of efficient crosstalk between the synapse and neighboring astrocyte processes and the extracellular matrix (ECM) [4,5]. Notably, both the pre- and postsynapse is surrounded by specific ECM constituents, which are anchored to the neuron’s cytoskeleton by a series of adhesion proteins that mediate the exchange of information between the inside and outside of the neuron and thereby actively participate in regulating plastic changes at synapses [6,7]. Importantly, both neurons and astrocytes use proteases anchored to the membrane or secreted into the perisynaptic space to actively control transsynaptic adhesion and shape the structure and composition of the ECM. Initially, extracellular proteases were believed to operate as brain ECM “movers,” but presently, they are known to selectively cleave defined adhesion proteins and structural or signaling molecules within ECM to trigger specific signaling pathways that regulate the physiology of the synapse during learning [8]. In addition, an essential role of extracellular proteases in pathological plasticity that is related, for example, to addiction [5], epilepsy [9], or Alzheimer’s disease [10] has recently been reviewed.

The family of matrix metalloproteinases (MMPs) consists of more than 20 proteolytic enzymes belonging to the metzincin superfamily. In terms of homology and substrate specificity, MMPs are divided into several subgroups, including collagenases (MMP1, 8, 3), stromelysins (MMP3, 10, 11), gelatinases (MMP2, 9), and proteases anchored in the cell membrane (MMP14, 15, 16, 17, 24, 25). Among all MMPs encoded in mammalian genomes, the majority of them was detected at the level of mRNA or protein in the healthy brain or in numerous pathological conditions [10,11]. All MMPs are synthesized as inactive zymogens (proMMPs) with a propeptide sequence blocking the catalytic domain. In addition, most MMP family members also contain a proline-rich hinge region and the hemopexin domain, which is responsible for interaction with other proteins, substrate recognition, binding of endogenous inhibitors, and proper localization in the extracellular space [12]. Even though not all MMPs are equipped with transmembrane domains, most of them operate in the vicinity of the cell surface by associating with membrane proteins that concentrate active forms of MMPs. Additionally, the proteolytic activity of MMPs is temporally and spatially restricted by tissue inhibitors of metalloproteinases (TIMP1, 2, 3, 4), with TIMP1 mainly blocking the activity of MMP2, 3, 9, and TIMP3 of almost every metzincin [13]. Finally, as with the majority of extracellular proteases, MMPs are internalized upon binding to the LRP1 (low-density lipoprotein receptor-related protein 1) [14]. For a detailed description of MMP molecular biology, with the emphasis on MMP9, we direct the reader to excellent reviews by Gomis-Rüth [15] and Vandooren et al. [16].

As already mentioned, due to the activity of MMPs, the synaptic adhesome and the extracellular matrix constituents are processed continuously upon induction of synaptic plasticity and, consequently, MMP-dependent proteolysis actively participates in both the structural remodeling of synaptic structures and functional changes in synaptic efficacy [5,17,18]. MMPs, secreted and activated upon increased neuronal activity, can locally loosen ECM to define hotspots of structural plasticity on a fragment of dendritic tree [17]. Simultaneously, active MMPs may activate other proteins through proteolysis, release new peptides from full-length proteins, and process adhesion molecules to trigger or terminate intracellular signal transduction pathways that would modulate the abundance of synaptic receptors and gene expression in the nucleus. Therefore, MMP-mediated proteolysis, with its irreversible nature, is an excellent candidate for a mechanism that ties the functional and structural component of synaptic plasticity to produce long-lasting changes underlying cognitive processes.

In this review, we discuss recent studies elucidating the roles of different MMPs in multifarious forms of synaptic plasticity, learning, and memory. Since MMP9 is the most widely studied MMP in the brain, we briefly outline its perisynaptic activation, interaction with local structures, and endogenous inhibition to explore the current knowledge on its role in neuroplasticity phenomena and cognitive processes in this context. Additionally, special attention is given to the latest studies investigating the role of MMP3 in molecular mechanisms governing the excitatory synaptic plasticity that depends on l-type calcium channels [18]. For years, research into the inhibitory synaptic plasticity was overshadowed by investigations into the plasticity of excitatory synapses, but this situation started to change a decade ago or so. In this report, we thus take advantage of recent progress in studies on mechanisms of GABAergic synaptic plasticity. A particular emphasis is placed on the key role of MMP3 in this type of plasticity, which was investigated by our group systematically in the hippocampus [19]. Finally, based on the latest findings, we review and discuss the increasing number of behavioral studies linking the activity of specific MMPs in excitatory and inhibitory neuronal networks to learning and memory.

## 2. Regulation of Synaptic Plasticity in Excitatory Synapses and Neuronal Excitability by MMPs

### 2.1. Background Considerations on the Involvement of MMPs in Synaptic Plasticity

Early evidence that metalloproteinases might play a central role in synaptic plasticity was derived from experiments on an epileptic mouse model, which showed elevated MMP2 and MMP9 in the hippocampus a few hours after injection of kainate [20]. Previously, the T. Bliss group reported increased proteolytic activity in hippocampal homogenates after LTP induction in perforant path synapses [21]. In particular, they described an increase in the activity of 80 kDa protease, which is capable of digesting the gelatin in gel zymography which can now be, with high confidence, ascribed to MMP9 [22]. In the follow-up study, the same group reported increased extracellular proteolytic processing of amyloid-beta precursor protein (APP) and neural cell adhesion molecule (NCAM) after LTP [23]. These results, together with the discovery of the role of tissue plasminogen activator (tPA) [24] and TIMP1 in LTP [25], provided the first direct evidence that elevated extracellular proteolysis can be a hallmark of long-term synaptic plasticity.

Having established the involvement of proteolysis in the phenomena of synaptic plasticity, the major question to be answered was which specific proteases were involved. Unfortunately, this issue proved difficult as a precise identification of MMPs involved in plastic changes is hampered by the lack of specific inhibitors of individual MMPs and compensatory effects in mouse gene knockouts. Indeed, available MMP inhibitors are not fully specific and selective, and they usually block two or more metzincins with a similar affinity. Thus, for this reason, it is advisable to use a wide battery of different inhibitors with a non-overlapping spectrum of blocked proteases to identify the involvement of individual MMP in the studied process and to exclude other proteases concurrently. Additionally, studies using genetic knockout animals should validate the synaptic role of a given protease, but caution should be exercised because of compensatory changes that may occur in MMP-deficient mice. For example, MMP9 deficiency leads to a compensatory increase in MMP8 expression [26]; similarly, MMP3 genetic knockout mice show elevated MMP7 and MMP12 [27]. Furthermore, genetically modified mice created using 129-derived embryonic stem cells often contain passenger mutations in sequences flanking targeted genes, hence contaminating the phenotypic outcome of genetic manipulation. For example, knockouts of several MMP genes located on mouse chromosome 9 (mmp3, mmp7, mmp8) contain passenger mutation that inactivates the gene encoding caspase 11, which is also a protease [28]. As a result, partial inactivation of caspase 11 may confound phenotypic interpretation of MMP genetic knockout models or perturb the identification of MMP substrates by mass spectrometry-assisted degradomic studies. Importantly, for some strains of MMP9 genetic knockout mice, the residual leakiness at the protein level was reported [29]. This problem, especially in the field of neuroscience, was not commonly realized until recently and it would be advantageous to confirm previous results on animal strains in which MMP (especially MMP9 which was shown to be problematic with this respect) deficiency has been unequivocally confirmed. Taking into account the important evidence presented by de Bruyn et al. [29], it seems necessary to accept as a prerequisite in these studies to demonstrate by means of e.g., gelatin gel zymography, that knockout mice do not show any gelatin zymolytic MMP9 levels whatsoever (aside from WT controls).

Given the multitude of proteins involved in synaptic plasticity, specific criteria were proposed to determine whether a given protein plays an active role in the molecular mechanisms of plasticity maintenance. When exploring the potential role of a new protein in synaptic plasticity or learning, three types of tests should be used, as it was summarized initially by John Lisman [30]. The first and the most common test, referred to as the necessity test, determines whether a protein is required for plasticity by pharmacological inhibition or genetic knockout. Next, the occlusion test, in which the active form of investigated protein is introduced, and the effect should occlude the induction of synaptic plasticity. Finally, the erasure test examines whether manipulation of analyzed protein at some time point after the induction of synaptic plasticity can reduce its maintenance [30]. We use these experimental criteria to summarize available information on the roles of specific MMPs in the molecular mechanisms of neuroplasticity.

Among all metalloproteinases, the MMP9 is the most extensively studied in the context of synaptic plasticity, learning and memory [31]. MMP9-deficient mice show impaired LTP in the hippocampal CA3-CA1 [18,32] and mossy fiber-CA3 projections [33,34] as well as in projections within the amygdala from the lateral to the basal nucleus and from the basal to the medial section of the central amygdala [35]. Furthermore, the administration of the active form of MMP9 spontaneously increases the efficacy of excitatory synaptic transmission [32,36] and occludes the induction of LTP [33,37]. In addition, the administration of MMP9 inhibitor up to 30 min after LTP induction disrupts the maintenance phase of induced plasticity [38,39]. Altogether, MMP9 passed the necessity, occlusion, and erasure tests, which explicitly proved the engagement of this protease in the consolidation phase of long-term potentiation.

### 2.2. The Role of MMP9 in LTP

#### 2.2.1. The Mechanism of MMP9 Synthesis and Release in the Perisynaptic Environment

In general, the functioning of metalloproteinases in the synaptic and perisynaptic space can be divided into the following well-defined stages: (1) The induction of synaptic plasticity caused by a temporary change in the neural network activity leads to the synthesis and secretion of a protease into the extracellular space. (2) Once activated and properly located, it cleaves other proteins and peptides according to its substrate spectrum. (3) After a short period of activity, a protease can be inactivated due to an autocatalytic mechanism, blocked by endogenous inhibitors or internalized through the interaction with membrane receptors. The role of MMP9 in excitatory synapses is described in reference to these three stages.

The mRNA encoding MMP9 is transported into dendrites, where it is locally translated, and the protease is released into the perisynaptic area in an activity-dependent manner [40]. MMP9 mRNA is a part of the FMRP (fragile X mental retardation protein) complex that controls its dendritic transport and translation [41]. Additionally, miR-131, an activity-regulated microRNA, binds to the 3′ untranslated region of MMP9 mRNA and negatively regulates its expression in neurons. Stimulation of excitatory synapses (e.g., by activating mGluRs) drives MMP9 translation due to the dissociation of miR-131 from polyribosomes [42]. Next, after the synthesis, the zymogen of proMMP9 is stored in Golgi-derived vesicles associated with the dendritic cytoskeleton [43].

The molecular mechanisms of MMP9 secretion into extracellular space upon increased neuronal activity are unclear. Therefore, future studies are needed to describe this process precisely, as was the case, for instance, with BDNF [44,45]. Nevertheless, at this point, it is important to note that MMP9-deficient mice show normal long-term depression (LTD) at excitatory synapses in the hippocampal CA1 field [32], suggesting that the frequency of synaptic stimulation or the timing of postsynaptic action potentials determines whether MMP9 is involved in a given type of long-term synaptic plasticity. Moreover, calcium influx through the NMDA receptor appears to be involved in MMP9 secretion because when NMDARs are blocked, the residual LTP recorded in the hippocampal CA1 is insensitive to MMP9 inhibition [18], and the endogenous protease is not activated [37].

The exocytosis of postsynaptic MMP9-containing vesicles occurs at membrane localizations lateral to the postsynaptic density either within or outside dendritic spines [46]. It is interesting how far from the synapse proMMP9 is released and how distant its diffusion is following activation during the induction of synaptic plasticity. The hemopexin domain of MMP9 is known to play a role of a multifunctional ligand for numerous membrane receptors and ECM proteins, such as β5-integrin, Ku70/Ku80 heterodimer, LRP1, chondroitin sulfate proteoglycans [12,47], or CD44 [48]. These interactions are expected to limit the diffusion process favoring the concentration of this protease in the vicinity of synapses. MMP9 is well-known to participate in the mechanism of structural plasticity of dendritic spines [49] and control the enlargement of small, but not large, dendritic spines in neuronal cell cultures after LTP induction [50]. However, the mechanisms underlying this selectivity are not clear: proMMP9 could be released selectively at small synapses prone to structural plasticity, or big spines could lack specific receptors for MMP9 that would concentrate it, enabling the protease to trigger the plasticity mechanisms.

#### 2.2.2. The Mechanisms of MMP9 Activation

**Figure 1:**
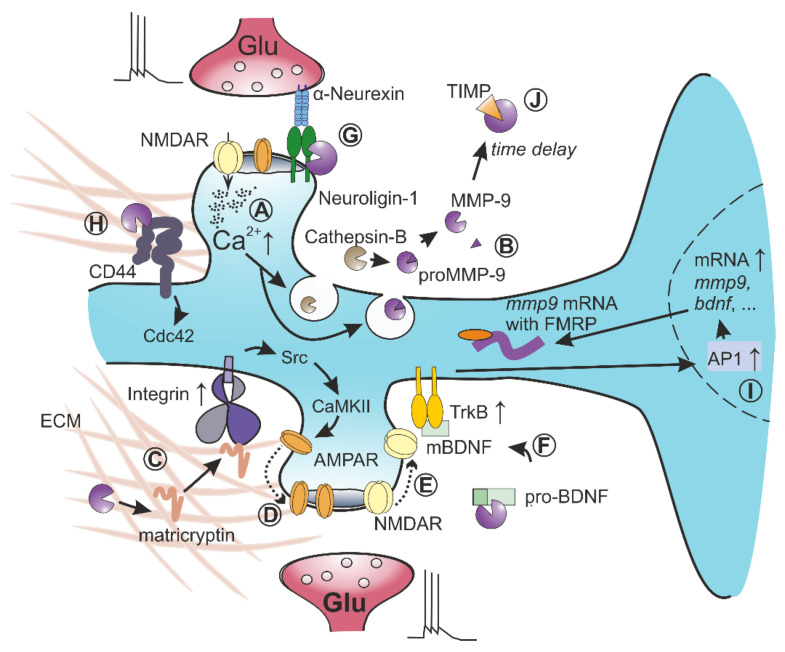
Functions of MMP9 in long-term potentiation at excitatory synapses. (**A**) High frequency or spike-timing-dependent stimulation leads to calcium influx in dendritic spine that triggers the exocytosis of lysosomes and secretory vesicles with proMMP9. (**B**) In extracellular space, proMMP9 becomes activated by lysosomal cathepsin-B. (**C**) Active MMP9 cleaves unidentified ECM constituents to release matricryptins, which activate integrins containing β1 subunit. (**D**) Integrin-dependent signaling pathways activate kinase Src and CaMKII to change the lateral diffusion of AMPA receptors, which results in their trapping at synapses and LTP induction. (**E**) Simultaneously, MMP9 activity increases the membrane diffusion of NMDA receptors that probably results in an alteration of the subunit composition of synaptic NMDARs. (**F**) Active MMP9 may cleave proBDNF to its mature form, which activates TrkB receptor in an autocrine manner. (**G**) Additionally, the proteolysis of neuroligin-1, transiently destabilizes synaptic adhesome during the induction of LTP. (**H**) CD44 anchors brain ECM to neuronal membrane through hyaluronan binding; MMP9-dependent cleavage of CD44 activates neuronal cdc42 that is crucial for structural plasticity of dendritic spines. (**I**) Signaling pathways activated during LTP induction reach the nucleus to induce the expression of immediate early genes, e.g., a c-fos, which give rise to appearance of the AP1 transcription factor and drive the transcription of mmp9 and bdnf genes among others. Their mRNA is transported to dendritic tree in a complex with FMRP protein. (**J**) After a short period of proteolytic activity, active MMP9 is blocked by endogenous inhibitor form TIMP family.

Once released into the extracellular space, brain MMP9 proform is activated by at least two different processes: (1) the proteolytic removal of inhibitory prodomain that in vitro can be accomplished by several proteases, such as tPA, uPA (urokinase-type plasminogen activator), MMP3 [16,51], or (2) through S-nitrosylation, which requires the synthesis of nitric oxide [52,53,54,55]. Nevertheless, the exact mechanism of MMP9 activation in the perisynaptic region in vivo or ex vivo in brain slices is far from being understood. The activation by MMP3 is rather unlikely because the effects of MMP3 and MMP9 inhibition on LTP induction do not occlude reciprocally [18]. However, an interesting mechanism of MMP9 activation in neurons was discovered by Padamsey et al. [56]. It was shown that the backpropagation of action potentials in a postsynaptic neuron elicited calcium influx that caused the secretion of the content of lysosomes. Consequently, lysosomal proteases, e.g., cathepsin B, were released into extracellular space, where they activated proMMP9 required for long-term structural plasticity of dendritic spines in hippocampal CA1 neurons [56]. Additionally, the activation of serotonergic receptors 5-HT7R stimulated the activity of MMP9 in the vicinity of dendritic spines and shafts in neuronal cultures [57]. Future studies should shed more light on the timing of secretion and spatial localization of exocytosis spots for lysosomes and proMMP9-containing vesicles during long-term synaptic plasticity (Figure 1B).

#### 2.2.3. Putative Targets of MMP9 Proteolytic Activity in the Perisynaptic Area

Active MMP9 in extracellular space can cleave numerous substrates that act as adhesion receptors, soluble signaling proteins, or structural ECM constituents (we refer the readers to excellent reviews on this topic [58,59,60]). However, considering the low specificity and pleiotropic nature of MMP inhibitors, we focus on neuronal MMP9 substrates validated in MMP9-deficient animals in vivo or ex vivo in brain slices.

A majority of well-documented synaptic substrates of MMP9 belong to different families of adhesion proteins. During the critical period, vision restoration after monocular deprivation induces massive synaptic plasticity in the visual cortex, accompanied by an increase in MMP9 activity [61]. This plasticity requires MMP9-dependent proteolytic processing of adhesion protein, neuroligin-1 (NLGN1), in a process that relies on NMDA receptors and CaMKII [62]. Similarly, stimulation of synaptoneurosomes with NMDA results in the MMP-dependent cleavage of neuroligin-1 [63]. This process destabilizes the transsynaptic neurexin-neuroligin complex, reducing the frequency of miniature EPSCs, presynaptic probability of neurotransmitter release, and the amplitude of evoked EPSC [62]. These results support a model where the time-limited extracellular proteolysis of NLGN1 transiently disrupts synaptic adhesive apparatus that leads to functional changes in the excitatory synapses and structural remodeling of the dendritic spines (Figure 1G). The crucial role of MMP9 in the timing of the visual critical period was also recently observed in a study in which astrocytic connexin signaling decreased the expression of MMP9 through RhoA signaling. This process resulted in the stabilization of perineuronal nets and the closure of a critical period in visual cortex [64]. This observation underscores the potential of both astrocytic and neuronal MMP9 in controlling plasticity phenomena in the developing brain.

It was demonstrated that MMP9 can also cleave and activate proBDNF to its mature form (mBDNF). The downregulation of mBDNF was observed in MMP9-deficient mice after kindling-induced epilepsy [65]. Additionally, during development and upon learning, the strengthened synapses are often co-arranged on the same dendritic branch. This synaptic clustering in hippocampal CA3 neurons requires MMP9 activity and mBDNF production that activates TrkB receptors [66]. It is noteworthy in this context that the enriched environment that stimulates massive synaptic plasticity also upregulates MMP9 and mBDNF in the hippocampus [67]. Moreover, a positive feedback loop was proposed in which mBDNF activates the TrkB receptor, that through ERK signaling stimulates formation of AP-1 transcription factor, and finally leads to MMP9 synthesis [68]. Altogether, these observations may indicate that MMP9 cleaves proBDNF to drive structural plasticity and proMMP9 restocking (Figure 1F).

In line with the crucial functions of MMP9 in the structural plasticity of dendritic spines, recent work demonstrated a key role for MMP9-dependent cleavage of CD44 in this process [57]. In this study, the stimulation of 5-HT7 receptors upregulated MMP9 activity, which resulted in CD44 cleavage and the activation of Cdc42 signaling that finally triggered dendritic spine remodeling and synaptic pruning (Figure 1H). Given the reported link between MMP9 and CD44, it is of note that CD44 operates as a receptor for hyaluronan, the main constituent of brain ECM and perineuronal nets. Significantly, while Fmr1 genetic knockout mice, a model of fragile X syndrome, are characterized by elevated MMP9 activity and loss of perineuronal nets around parvalbumin-expressing interneurons in the auditory cortex of juvenile mice, the MMP9 deficiency restored the proper PNN formation [69]. Additionally, light reintroduction after dark exposure can trigger a massive degradation of ECM accompanied by changes in neuronal excitability, synchrony, and enhanced structural plasticity of dendritic spines. These effects are blocked by genetic ablation of MMP9, indicating that increased activity of this protease reduced constraints on structural and functional plasticity in the mature cortex [70,71]. In this regard, while additional studies are needed to elucidate the impact of MMP9 on ECM development fully, it is increasingly evident that MMP9 activity is intimately associated with ECM macroscopic integrity [64,72].

Interestingly, in most cases in which MMP9 was implicated in the plasticity of excitatory synapses, its action required integrin β1 in the hippocampus or cortex [11,33,72] or integrin β3 in nucleus accumbens [73]. Indeed, the potentiation of synaptic transmission after treatment with active MMP9 is completely abolished by integrin inhibitors [32] or by antibody blocking integrin β1 functions [36]. Furthermore, it was also shown that active MMP9 applied exogenously increased the lateral diffusion of NMDA receptors containing GluN1 subunit, both in synaptic and extrasynaptic localization, through a process that is blocked by β1 integrin inhibition [74]. Additionally, the application of exogenous active MMP9 caused changes in the morphology of dendritic spines, and this process also depends on functional β1 integrins whose signaling drives cofilin phosphorylation and actin polymerization [36,75].

One of the most critical questions in the research on the role of MMP9 in synapses is the identification of MMP9 substrates, which, after cleavage in perisynaptic space, can activate β1 integrin (Figure 1C). The term matricryptin was proposed to describe peptides resulting from extracellular proteolysis that show new properties compared to full-length parent protein [76]. In this scenario, extracellular proteases decode and unveil the hidden cryptome of adhesion and ECM proteins. However, the identification of matricryptins released by MMP9 is still a matter of debate. It was shown that the extracellular domain of postsynaptic adhesion receptor ICAM-5 (a well-known MMP9 substrate) might bind and activate presynaptic β1 integrin in cultured hippocampal neurons [77], but this process is unlikely to be responsible for LTP maintenance as mature spines lack ICAM-5 [77,78]. Thus, it seems that ICAM-5 is important for synaptogenesis and spine maturation [79,80] rather than plasticity at mature synapses.

Overall, new findings concerning the role of β1 integrins in synaptic plasticity would also shed some light on the functioning of neuronal MMP9. For example, interference with the integrin β1-dependent adhesion, similar to MMP9 inhibition, blocked the long-term structural plasticity of dendritic spines and the reorganization of actin microfilaments inside spines in hippocampal slices [81]. In addition, after the induction of LTP in the hippocampus, integrin β1 became activated only for a few minutes, shortly after the application of the plasticity-inducing protocol [82]. This result suggests that MMP9, located upstream in the signaling pathway, also should be activated at early stages of LTP, which agrees with the time window of MMP9 requirement for LTP, inferred from pharmacological experiments [38,83]. This also implies that the presence of active integrin β1 in the synapse may be regarded as a marker of recent MMP9 activity. The comparison of the time scale of synaptic transmission with the timing of MMP9 involvement in plastic changes within synapses is intriguing. While millisecond precision is required for the induction of spike-timing-dependent plasticity at excitatory synapses [84], it takes a few minutes from the application of LTP induction protocol to MMP9 release and activation [32]. Then, after 10–20 min, the protease undergoes endogenous inhibition and/or internalization [85]. Figure 1 schematically summarizes the synaptic functioning of MMP9.

The development of high-throughput degradomic techniques has resulted in the discovery of many potential MMP9 substrates in different tissues in physiological and in pathological conditions [86,87,88]. Among putative MMP9 substrates described in this way, several are expressed in the brain, where they can be found in intracellular, membrane or extracellular compartments of the synapse. Tumor Necrosis Factor-α (TNFα) is noteworthy [86] as it was shown to control synaptic efficacy [89] and to regulate homeostatic plasticity [90]. Similarly, MMP9 may cleave and activate ADAM Metallopeptidase With Thrombospondin Type 1 Motif 4 (ADAMTS4) [86], a metzincin able to cleave brevican during homeostatic synaptic up-scaling [91]. These two examples show that among putative MMP9 substrates discovered in vitro outside the brain, many may play an important role in synapse physiology and during synaptic plasticity but these scenarios still remain to be demonstrated. Finally, neuronal MMP9 may cleave and degrade β-amyloid peptide in brains of patients suffering from the Alzheimer’s disease [92]. Interestingly, endogenous amyloid-beta peptide controls presynaptic release probability in healthy brain [93], suggesting that putative MMP9-dependent degradation may also control presynaptic neurotransmitter release.

#### 2.2.4. The Crucial Role of Endogenous MMP9 Inhibition

After vesicular release followed by activation, MMP9 exerts its proteolytic activity for a relatively short time to be eventually inhibited, avoiding excessive proteolysis (Figure 1J). This endogenous inhibition is an essential step in MMP9 functioning and ensuing regulatory phenomena, as it was demonstrated that the outcome of the short-term MMP9 activity might dramatically differ from the effects of its long-term presence. For example, the brief treatment with active MMP9 followed by protease inhibition or washout 10–20 min later increased the efficacy of CA1 excitatory synapses [32] and induced changes in the morphology of dendritic spines toward mushroom-like shape [36,85]. In contrast, the long-lasting presence of exogenous MMP9 or its overexpression caused the transformation of dendritic spines into a filopodial shape [75] and impaired LTP [34,83,84]. The crucial role of MMP9 inhibition in its synaptic functioning is further supported by the evidence of enhanced LTP in slices isolated from mice treated with intravenous injections of tissue inhibitor of metalloproteinases-2 (TIMP2) [94]. Additionally, the involvement of endogenous MMP inhibition in neuronal plasticity and learning is further confirmed by spatial memory deficits in TIMP3-deficient mice [95], impaired fear-potentiated startle response in TIMP2-null mice [96], and impaired LTP observed after TIMP1 overexpression in the prefrontal cortex [97].

Increased level of MMP9 activity was reported in numerous brain regions after inducing long-term plasticity and after learning. For example, the proteolysis mediated by MMP9 is augmented in the perisynaptic region of hippocampal neurons after LTP induction in different projections [32,36,98]. Similarly, increased gelatinolytic activity, ascribed to MMP9, was reported after contextual fear conditioning [99], after classical conditioning in a paradigm of pairing whisker stroking with tail shock [84], after chronic restraint stress [100], after a period of breeding in an enriched environment [101], or after appetitive learning [102]. Moreover, learning-induced augmentation of MMP9 activity in a considered brain region can be constrained to specific types of neurons. For example, learning in cue-induced heroin seeking paradigm increased MMP9 activity only around D_1_ receptor-expressing medium spiny neurons in the nucleus accumbens, while subsequent extinction training augmented MMP2 activity around contiguous D_2_ medium spiny neurons [103]. This compartmentalization of the involvement of MMP9 and MMP2 in synaptic plasticity suggests that different neurons could, in principle, express and use distinct MMPs during plastic changes occurring at the synapse.

In physiological conditions, the relative timing of MMP9 activation and the secretion of TIMPs during LTP or learning is unknown. Interestingly, both proteins were spotted in the same dendritic secretory vesicles in vitro [43], suggesting that the time window of MMP9 activity may not be determined by the delayed release of TIMPs. Additionally, the requirement of short-lasting MMP9 activity upon LTP induction, inferred from pharmacological experiments [38,83], appears to disagree with the observation of increased MMP9 activity for hours after LTP induction or even days after learning. This apparent inconsistency may be explained by the limitations of in situ gelatin zymography, the most common technique used to visualize MMP9 activity in the tissue. Before staining, the tissue is usually fixated in alcohol (usually methanol with ethanol) that reversibly denature proteins which are then renatured during the stage of hydration [22]. This process disrupts the interaction of MMP and TIMP. Consequently, after the fixation in alcohol, the active form of MMP9 that is blocked in the tissue by TIMP may dissociate from the inhibitor and show in situ activity. Similarly, during the procedure of tissue homogenization before gelatin gel zymography, the complexes of active MMP9 and TIMP are broken; therefore, the intensity of gelatinolytic band ascribed to active MMP9 may not precisely correspond to the level of MMP9 activity in the tissue [22]. In summary, the active MMP9 assessed using zymography corresponds to the combined level of the protease that is indeed active in the neuropil and the component of MMP9 that dissociates from TIMPs due to in vitro processing. Additionally, natural proMMP9 occurs as monomers or stable homotrimers with different binding affinity to TIMP1 [104] and distinct clearance mechanism [105]. Future studies should shed more light onto the role of trimeric MMP9 and its inhibition in the brain.

#### 2.2.5. MMP9 in Learning

Given the unique MMP9 function in synaptic plasticity, significant efforts were undertaken to elucidate MMP9 roles in different types of learning and memory. Again, because of the low specificity of available inhibition or insufficient testing against a wide range of MMPs, we limited our considerations to studies in which genetic knockout models were used. Early work in mice showed that MMP9-deficient mice are characterized by impaired contextual fear conditioning that depends on the cortex and hippocampus [32,106]. Additionally, Nagy et al. observed normal amygdala-dependent cued fear conditioning in MMP9 mouse gene knockouts [32], whereas another group reported impaired learning in this paradigm [106]. The unique MMP9 function in the amygdala may explain this discrepancy, where it is required for learning in the conditioning paradigms that use appetitive but not aversive motivation [102]. MMP9-deficient mice also show impairments in novel object recognition and decreased anxiety [106]. Moreover, MMP9 is also required for the reorganization of the somatosensory cortex after sensory deprivation [107] and for massive synaptic plasticity after light reintroduction in binocular adult mice [70,71].

In line with reported changes in gelatinolytic activity in different models of addiction [108], numerous studies demonstrated compromised substance abuse craving or relapse in MMP9-deficient models [109,110]. For example, injection of MMP9 inhibitor into the nucleus accumbens reduced cue-induced reinstatement of cocaine-seeking behavior [111]. Another report linked MMP9 function to alcohol addiction [112]. This study demonstrated that mouse MMP9 knockouts had decreased motivation for alcohol after withdrawal in a process that requires synapse silencing in the central nucleus of the amygdala.

Despite the crucial role of MMP9 in synaptic plasticity, learning, and memory, the high-throughput mass spectrometry proteomic studies aimed at finding learning-associated proteins rarely reported learning-driven up- or downregulation of MMP9. This peculiarity may be explained by a low level of MMP9 expression in the naïve brain [31] or the cell specificity of MMP9 operation. Interestingly, MMP9 was reported to be strongly upregulated in cortical somatostatin-containing interneurons in dark-housed mice after exposure to light that activates substantial cortical plasticity [113]. A similar line of research was recently pursued by Salamian et al., who demonstrated that in slice cultures, MMP9 inhibition impairs carbachol-induced plasticity in excitatory synapses on CA1 fast-spiking GABAergic interneurons [114]. This result opens new avenues for studies of MMPs in the context of excitatory synaptic transmission onto interneurons during learning.

### 2.3. The Role of MMP3 in LTP

**Figure 2:**
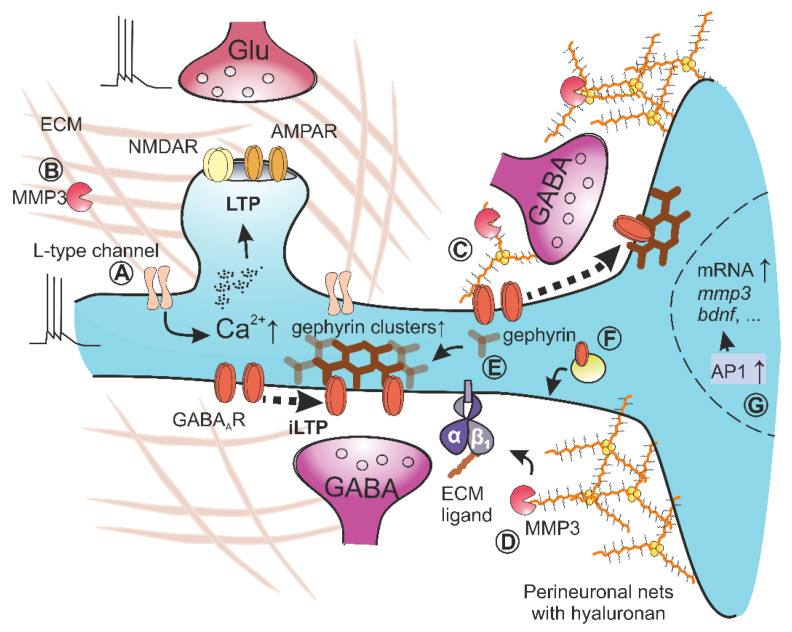
MMP3 in long-term potentiation at excitatory and inhibitory synapses. (**A**) High-frequency stimulation of excitatory synapse leads to calcium influx into the postsynaptic cell through L-type voltage-dependent calcium channels and NMDA receptors. (**B**) MMP3 by cleaving unknown ECM constituent controls l-type-dependent LTP but not NMDA-LTP. (**C**–**G**) Stimulation of excitatory synapses leads to the heterosynaptic long-term potentiation of nearby inhibitory synapses located on the dendritic tree and soma. (**C**) Active MMP3 cleaves unidentified ECM constituents to release matricryptins, which activate signaling pathways in a postsynaptic neuron, resulting in decreased lateral diffusion and increased trapping of GABA_A_ receptors at inhibitory synapses. This causes the induction of iLTP. (**D**) Activation of integrins containing β1 subunit potentiates the efficacy of inhibitory synaptic transmission. Matricryptins engaged in this process may be released by MMP3 from perineuronal nets. (**E**) MMP3 activity augments the size of synaptic clusters of gephyrin—the main structural protein of postsynaptic density at inhibitory synapses (**F**) iLTP induction is accompanied by increased exocytosis of GABA_A_ receptors. (**G**) Signaling pathways activated during a period of increased synaptic activity induce the transcription of new genes in the nucleus. The activity of c-fos, which forms the AP1 transcription factor, is required to drive the transcription of new mRNAs encoding proteins crucial for iLTP such as MMP3, proBDNF or different integrins.

Generally, LTP can be divided into two phases, the early one that depends on posttranslational modification of existing synaptic proteins and the late-phase that relies on transcription in the nucleus and translation of new proteins. Determining which LTP phase is affected by interference with a given protein thus provides essential information on its role in the signaling pathway underlying the plasticity. Interestingly, in studies addressing the involvement of MMP9 in plasticity phenomena, the extent of LTP impairment observed in MMP9-deficient mice [18,35] was found to show apparent differences with respect to LTP recorded in the presence of MMP inhibitors, such as FN439 or NNGH [32,36,83,85]. However, in studies in which different MMPs blockers were used, their impact on LTP showed differences, which eventually proved insightful. Indeed, some reports indicated the LTP impairment from the very beginning after induction in the presence of FN-439 or NNGH [36,39,83,115,116]. However, other studies in which more specific MMP9 inhibitors were used (e.g., SB-3CT, MMP9 inhibitor I or S24994) reported a reduction in the consolidation phase of LTP that started from ~1 h after induction in hippocampal CA3-CA3 or CA3-CA1 projections and the prefrontal cortex [32,35,37,97,117]. Subsequent studies attributed this discrepancy to the unspecific activity of commonly used MMP9 inhibitors, which blocked other MMPs engaged in synaptic plasticity. Indeed, early impairment of LTP in the presence of broad-spectrum MMP inhibitors was explained by the activity of two metalloproteinases, namely MMP9 and MMP3. While the classical form of LTP, which is dependent on NMDA receptor (NMDA-LTP), was compromised in MMP9-deficient mice, the form of LTP that requires the activity of L-type voltage-dependent calcium channels (VDCC-LTP) was abolished in MMP3-deficient slices [18]. The crucial role of MMP3 in LTP in the hippocampal CA1 region was subsequently confirmed in the necessity, occlusion, and erasure tests. MMP3-deficient mice showed impaired LTP in the hippocampal CA3-CA1 projection but not in mossy fiber-CA3 pathway [18]. Furthermore, the administration of the active MMP3 increased the glutamatergic synaptic currents [118]. In addition, the administration of MMP3 inhibitor up to 15 min after LTP induction disrupts the maintenance phase of induced plasticity [18]. Figure 2A,B presents schematically the current knowledge on the role of MMP3 in VDCC-LTP.

In comparison to NMDA-LTP, molecular mechanisms of VDCC-LTP induction and maintenance have not been thoroughly investigated. VDCC-LTP was shown to have a clear dependence on ECM integrity because digestion of hyaluronan, the main component of the brain’s ECM, completely abolished the induction of this form of LTP [119]. Interestingly, MMP3 inhibition did not affect LTP recorded in hippocampal slices treated with hyaluronidase that digests hyaluronan. It suggests that proteolysis of ECM elements by MMP3 promotes VDCC-LTP, possibly through the generation of new matricryptins. Moreover, evidence from follow-up studies lends further support for an association between ECM proteins and VDCC-LTP, as electrophysiological analyses in tenascins-C-deficient mice found impairment of this form of LTP [120]. Furthermore, tenascins-C can be cleaved by MMP3 [121], and this cleavage may act as a permissive factor upon induction of VDCC-LTP. Additionally, VDCC-LTP also requires nitric oxide synthesis that operates as a transsynaptic retrograde messenger [122,123], and nitric oxide may be necessary for activation of synaptic or perisynaptic MMP3. Finally, the proteases responsible for the activation of MMP3 in perisynaptic space are not known. In vitro, MMP3 pro-enzyme can be activated in extracellular space by the tPA/plasmin system [124].

MMP3 was also implicated in long-term plasticity in the cerebral cortex; however, differences between cortical regions were observed in this respect. In the somatosensory cortex, LTP induced by the spike-timing-dependent paradigm in synapses from layer V to II/III relies on MMP9 but not MMP3 [84]. In contrast, LTP induced with a similar protocol in the anterior cingulate cortex depends on MMP3 but not MMP9 [125]. It was also demonstrated that MMP3 deficiency led to impaired plasticity in the visual cortex after monocular enucleation [126]. In this study, aberrant neuronal morphology was reported in the visual cortex of MMP3-deficient mice [126]. Similar morphological impairments were described in the cerebellum [127] but not in CA1 hippocampal pyramidal cells [128].

While extensive MMP9 activity profiling was performed in different brain regions using in situ gelatin zymography, the activity of other MMPs may also be investigated using different fluorogenic substrates. For instance, casein in situ zymography carried out in the presence of serine protease inhibitors allowed for the visualization of MMP3 activity in the hippocampus [18]. Interestingly, this method showed that LTP induction led to increased MMP3 activity in the CA1 stratum radiatum. Notably, while MMP3 protein was detected in neurons and astrocytes, the active MMP3 colocalized with the marker of synapses, suggesting a prominent synaptic and perisynaptic locus of this protease when activated [18].

In the hippocampus of adult mice, the level of MMP3 mRNA is very low [124]. Nevertheless, changes in MMP3 expression were reported after learning. An increase in the level of hippocampal MMP3 mRNA and protein was observed during spatial learning in the Morris water maze [39]. Additionally, an elevated level of active MMP3 in the hippocampus was detected from 1 to 4 h after passive avoidance conditioning [129] and head-shake response habituation [130].

Additionally, some evidence suggests that MMP3 may also affect NMDA receptors. Massive calcium influx through NMDAR in cultured spinal cord neurons caused an increase in the activity of MMP3, which in turn cleaved the GluN1 subunit of the NMDA receptor and alleviated further calcium influx [131]. In addition, Brzdak et al. recently demonstrated that MMP3 controls long-term potentiation of NMDAR activity in apical but not basal dendrites of pyramidal neurons in the hippocampal CA1 region [118]. The activity of MMP3 is also crucial for the plasticity of neuronal excitability [116]. Nevertheless, additional research is needed to address the mechanism by which MMP3 controls neuronal excitability and its possible role in LTD.

### 2.4. Other MMPs in Synaptic Plasticity

Considering the multitude of MMPs expressed in the brain (reviewed in [10]), it is very likely that the current list of MMPs involved in synaptic plasticity is far from complete. Indirect evidence that points to the role of MMPs other than MMP9 and MMP3 in long-term synaptic plasticity comes from the studies of long-term depression. Despite the compromised LTD in the presence of a broad-spectrum MMP inhibitor FN439 in CA1 hippocampus [83], it is noteworthy that electrophysiological recordings in slices from MMP9-deficient mice showed unchanged LTD [32]. Additional experiments are required to identify protease necessary for LTD. Nevertheless, preliminary analysis of FN439 specificity shows that, at commonly used concentrations, this compound blocks—in addition to MMP9—MMP1, 2, 3, 8, and partially tumor necrosis factor-alpha converting enzyme (TACE), also known as ADAM17 [132,133]. Further evidence for the participation of unidentified metalloproteinases in the molecular mechanisms of synaptic plasticity comes from the study of the proteolysis of Netrin-G ligand-3 (NGL3). It is a postsynaptic adhesion protein that interacts with presynaptic receptor tyrosine phosphatases from the LAR family. Interestingly, NGL3 undergoes proteolytic processing mediated by unspecified metalloproteinase during the induction of mGluR-dependent LTD in hippocampal slices. The identity of responsible protease is not known, but the cleavage is blocked by a pan-MMP inhibitor GM6001 [134].

Epilepsy and seizures in the brain are often associated with hyperplasticity of synapses, changes in neuronal excitability, and massive morphological alterations (e.g., aberrant sprouting of mossy fibers). Several MMPs were upregulated in animal models of epilepsy, including MMP9 [135], MMP2, 14 [136], MMP3, and MMP13 [137]. It seems thus that in epilepsy not only some specific proteases are affected but rather an interdependent network of proteases is compromised. Similarly, the elevated expression of MMP1, 2, 8, 9, 10, and 13 was reported in epileptic patients [138]. Additionally, the mRNA of MMP2, 3, 7, 9, and 24 binds to the FMRP in dendrites and undergoes activity-dependent translation [139]. Among proteases listed above, synaptic functions were also found to be modified by MMP7. Administration of exogenous active MMP7 in cultured hippocampal neurons alters the morphology of dendritic spines towards elongated filopodia, indicative of immature spines [140]. Furthermore, MMP7 may cleave GluN1 and GluN2A subunits of the NMDA receptor diminishing calcium influx in hippocampal pyramidal neurons [141]. Thus, MMP7 may control the level of calcium in dendritic spine during synaptic plasticity and learning. However, the mechanism whereby MMP7 participates in these processes awaits detailed investigation. To conclude, the findings discussed so far emphasize the pleiotropic roles of MMPs in excitatory synapses and raise the interesting question regarding the identity of MMPs, which, in addition to MMP3 and MMP9, regulate long-term plasticity at the level of single synapses, neuronal excitability, and neuronal networks.

## 3. Extracellular Proteolysis in the Plasticity of Inhibitory Synapses

### 3.1. Synaptic Plasticity of Inhibitory Synapses

Cortical and hippocampal inhibitory interneurons comprise a heterogeneous group of neurons featuring diverse morphology, synaptic targets, and network properties [142]. This variety also becomes apparent at the level of GABAergic synapses that are either formed at excitatory principal cells or other interneurons [143]. Despite the numerous vital functions that synaptic inhibition performs during the development, in rhytmogenesis, or during learning, the rules and molecular mechanisms underlying the plasticity of GABAergic synapses remained elusive for decades. They only started to be unveiled recently, taking advantage of novel methods that allow tracking the activity of specific interneurons.

The most commonly used nomenclature divides all interneurons into three major groups that express the calcium-binding protein parvalbumin (PV), neuropeptide somatostatin (SST), and vasoactive intestinal peptide (VIP). In addition, these groups are subdivided based on the coexpression of other markers (like cholecystokinin CCK or neuropeptide Y) and electrophysiological or morphological properties. In recent years, inhibitory long-term plasticity in the form of iLTP (GABAergic inhibitory long-term potentiation) and iLTD (inhibitory long-term depression) was described in several brain regions, including the hippocampus, cortex, nucleus accumbens and cerebellum. For an in-depth review of GABAergic plasticity and its learning-related functions, we direct the reader to recent excellent reviews [144,145,146,147].

The long-term plasticity mechanisms at GABAergic synapses can be divided by considering the locus of expression as either pre- or postsynaptic. Presynaptic inhibitory plasticity is mainly associated with the changes in GABA release, and the underlying mechanism is most commonly specific to presynaptic interneurons. This plasticity requires the communication between the postsynaptic site and the presynaptic terminal through retrograde signals such as endocannabinoids or nitric oxide [148]. In many brain regions, repetitive afferent stimulation triggers the synthesis of endocannabinoids in the postsynaptic cell, their trans-synaptic diffusion to the presynaptic terminal to suppress GABA release in a short- or long-term manner [149].

Postsynaptic plasticity of inhibition depends either on the changes in Cl^−^ equilibrium or the dynamically regulated abundance of GABA_A_ receptors in the postsynapse. The density of synaptic GABA_A_ receptors and thus the efficacy of inhibitory transmission is determined by the balance between the exocytosis of receptors, their membrane lateral diffusion, synaptic trapping, and endocytosis. At GABAergic synapses, heterosynaptically induced iLTP or iLTD depends on a postsynaptic calcium influx through NMDA receptors altering synaptic immobilization of GABA_A_ receptors [150,151,152]. Similarly, homosynaptic forms of iLTP and iLTD that rely on postsynaptic calcium influx through T-type voltage-gated calcium channels were described [153,154,155]. Another type of postsynaptic GABAergic plasticity depends on the changes in the intraneuronal Cl^−^ concentration. In the adult brain, the concentration of intracellular chloride ions is low due to the extrusion of these anions by the KCC2 transporter. Increased neuronal activity can downregulate KCC2 leading to increased intracellular Cl^−^ and thus more depolarized Cl^−^ equilibrium potential. As a result, this leads to the reduced amplitude of GABAergic synaptic currents [156].

In comparison with excitatory synapses, GABAergic synapses contain a distinctive set of adhesion proteins that control synapse properties, development, and plasticity [157,158]. Additionally, the subset of inhibitory synapses, but not excitatory, is located on neuronal soma where exceptionally dense ECM structures, called perineuronal nets, are formed [17]. It suggests a putative involvement of PNNs in the inhibitory plasticity of GABAergic synapses located on neuronal soma. These features collectively support the hypothesis that GABAergic synapses may contain a unique set of extracellular proteases that modify synaptic adhesome and produce new matricryptins during the development and inhibitory long-term plasticity [150,153].

### 3.2. MMP3 in the Plasticity of Inhibitory Synapses

The first hint that MMPs play a role during plastic changes at inhibitory synapses came from studies aimed to identify alterations to the network of GABAergic interneurons in the piriform cortex after kindling-induced seizures [159]. Doxycycline, a broad-spectrum MMP inhibitor, prevented perineuronal nets breakdown and the reorganization of GABAergic innervation during kindling.

Recently, a crucial role of MMP3 in the regulation of inhibitory synapses was described [19]. Both the pharmacological inhibition and MMP3 genetic knockout abolished iLTP induced heterosynaptically through moderate activation of NMDA receptors. Interestingly, MMP3 affects excitatory LTP and inhibitory iLTP within different time windows. Whereas MMP3 is needed for LTP in hippocampal CA1 excitatory synapses up to about 25 min after 100 Hz stimulation [18], iLTP induced in the same region requires MMP3 activity only up to approximately 10 min after the plasticity induction [19]. This observation supports the view that MMP3 is involved in mechanisms controlling plastic changes at excitatory and inhibitory synapses, but the underlying mechanisms are most likely different. Several reports describe intraneuronal signaling associated with NMDA-iLTP. For example, an elegant study by Petrini et al. demonstrated that during the induction of NMDA-iLTP, GABA_A_ receptors are immobilized at postsynaptic density, thereby strengthening the synapse [151]. Indeed, after iLTP induction, synaptic but not extrasynaptic α1GABA_A_ receptors were characterized by slower and more constrained diffusion, and this immobilization was impaired in the presence of MMP3 inhibitor [19]. Additionally, the short-term application of active MMP3 induced synaptic trapping of GABA_A_ receptors, increased the size of gephyrin synaptic clusters, potentiated mIPSC amplitudes, and restored impaired iLTP in MMP3-deficient neuronal cultures (Figure 2E) [19]. Figure 2C–G presents schematically the current knowledge on the role of MMP3 in NMDA-iLTP.

Although this evidence indicates that MMP3 has passed the necessity, occlusion, and erasure tests required to classify this protease as plasticity-related protein, numerous open questions remain. First, while the administration of exogenous active MMP9 increased the diffusion rate of synaptic and extrasynaptic NMDA receptors [74], active MMP3 decreased diffusion of only synaptic fraction of α1GABA_A_ receptors (Figure 2C) [19]. Thus, the perisynaptic activity of these two proteases has an opposite effect on the lateral diffusion of key glutamate and GABA-activated receptors. This “asymmetry” of MMPs in acting on critical receptors mediating excitation or inhibition is very interesting, but the underlying mechanisms remain to be explained. Additionally, it was shown that a broad spectrum metzincin inhibitor, GM6001, blocked LTP-driven immobilization of synaptic GluA1 containing AMPA receptors [50], similar to the way that MMP3 inhibitor suppressed α_1_GABA_A_Rs immobilization at inhibitory synapses upon induction of NMDA-iLTP [19]. Second, in comparison to MMP9, which mainly cleaves membrane adhesion proteins, MMP3 has a distinct substrate repertoire as it processes primarily ECM constituents such as collagens, fibronectin, laminin, osteopontin, tenascins, and all brain proteoglycans, thereby producing biologically active matricryptins form ECM (reviewed in [124]). A common finding of numerous studies is that digestion of perineuronal nets, for example, with chondroitinase, decreases GABAergic inhibition [160,161]. At the same time, augmentation of PNN abundance by rearing mice in an enriched environment increases synaptic inhibition [6]. This phenomenon is likely regulated by matricryptins produced by MMP3 as, for instance, a cleavage product of collagen XIX was found to promote the formation of inhibitory synapses in cultured hippocampal neurons [162]. Similarly, peptides containing RGD sequence enhanced GABA tonic current in the dentate gyrus [163] and modified inhibitory glycinergic transmission in cultures of spinal cord neurons [164]. Third, experiments with MMP3 genetic knockout models indirectly indicated that induction of iLTP was accompanied by changes in the kinetics of inhibitory synaptic currents [19]. This phenomenon may be due to the impact of MMP3-dependent signaling on the different mechanisms underlying regulation of GABA_A_Rs and GABAergic currents [163,165]. Finally, likewise MMP9, the expression of MMP3 is also driven by AP-1 transcription factor formed by, e.g., a c-fos protein that is expressed as neuronal immediate early gene [166]. Recently, it was shown that the c-fos transcription factor in pyramidal cells drives the expression of learning-related genes that modify the efficacy of inhibitory synaptic transmission in hippocampal CA1 (Figure 2G) [167]. Future studies should elucidate whether AP-1 driven expression of MMP3 is an endogenous factor involved in shaping perineuronal nets during GABAergic plasticity. A novel aspect of MMP3 in regulating the features of neuronal networks is the observation that deficiency of this protease caused a transient delay in migration of cerebellar interneurons and formation of inhibitory synapses [127]. Moreover, in vivo administration of an MMP inhibitor impaired the positioning of GABAergic interneurons in superficial cortical layers [168].

Given the crucial role of MMP3 in iLTP at inhibitory synapses, we checked how the genetic deletion of this protease affected memory and learning. Intriguingly, behavioral studies of adult MMP3-deficient mice showed enhanced spatial learning in the Morris water maze and increased fear conditioning by spatial context, but not by an auditory cue [19]. This phenotype contrasts with learning impairments reported in animals treated with MMP inhibitors that also block, among other proteases, MMP3. For instance, infusion of MMP3 inhibitor I before passive avoidance conditioning significantly impaired this type of fear learning [129]. Similarly, FN-436, a broad-spectrum MMP inhibitor that blocks MMP3 and MMP9, compromised the Morris water maze learning [39]. It is possible that considering the aforementioned aspecific action of MMPs blockers, these cognitive impairments observed in experiments in which MMP inhibitors were administered might result from blockade of proteases other than MMP3.

### 3.3. MMPs in Inhibitory Synapses and Their Putative Substrates

Inhibitory synaptic transmission is crucial for brain rhytmogenesis [169]. In brain slices, neuronal activity in the gamma band can be induced experimentally by carbachol. Interestingly, venlafaxine, an antidepressant from the family of monoamine reuptake inhibitors, was found to upregulate the power of carbachol-induced gamma activity in wild-type slices but not in those from MMP9-deficient animals [170]. This observation suggests that the activity of MMP9 promotes the augmentation of gamma rhythms that are usually decreased in patients with severe depression and restored during remission of depressive symptoms [171]. Two possible scenarios can explain this effect: MMP9 may regulate excitatory synapses onto interneurons or affect the efficacy of inhibitory synapses [114] to precisely control the fine balance between excitatory and inhibitory synaptic transmission. Notably, carbachol treatment in hippocampal slice cultures upregulated the frequency and the amplitude of miniature inhibitory synaptic currents (mIPSC) in pyramidal neurons and the frequency of excitatory mEPSC onto fast-spiking interneurons in a MMP-dependent manner [114]. Thus, although MMP9 is not required for NMDA-induced iLTP in pyramidal neurons of CA1, it cannot be excluded that MMP9 might be involved in other types of heterosynaptic GABAergic plasticity or plastic changes at excitatory synapses on GABAergic interneurons.

An adhesion protein, β-dystroglycan, is one of the best-documented MMP (especially MMP9) substrates processed upon induction of synaptic plasticity and learning [99,172]. Interestingly, brain dystroglycan localizes specifically at inhibitory synapses or astrocytes in the hippocampus or cortex [173,174]. Neuronal staining against dystroglycan appears limited to inhibitory synapses formed by CCK-interneurons [175]. Moreover, conditional deletion of the gene encoding dystroglycan nearly abolished the formation of inhibitory synapses by CCK-positive interneurons in the hippocampal CA1 region but left unchanged other inhibitory synapses [175]. It was suggested also that dystroglycan regulates GABAergic homeostatic plasticity. Indeed, chronic elevation of neuronal activity upscales inhibitory synaptic transmission in a mechanism that requires dystroglycan binding to ECM constituents [176]. Additionally, dystroglycan-deficient mice showed an augmented induction phase of excitatory long-term potentiation in the CA1 region of hippocampal slices, possibly due to reduced GABAergic activity [177]. While applied exogenously, active MMP9 might cleave dystroglycan in all extracellular locations, in physiological conditions MMP9 was not present at inhibitory synapses [135,178], and MMP9-deficient mice had an unchanged level of inhibitory synapses in the hippocampus [179]. These results thus suggest that MMP9 might cleave dystroglycan in astrocytic processes in the vicinity of synaptic structures rather than within inhibitory synapses. Thus, the identity of metalloproteinase responsible for β-dystroglycan processing which is located at inhibitory synapses awaits further studies.

Long-term regulation of synaptic transmission by BDNF was also observed at GABAergic synapses [180]. At excitatory synapses, proBDNF is processed into a mature form by MMP9 or tPA/plasmin. However, the protease responsible for the conversion of proBDNF into mature form at inhibitory synapses remains unknown.

Neuroligin-2 (NLGN2), the best-known adhesion protein at inhibitory synapses, is located specifically at the postsynaptic density, where it interacts with scaffold protein gephyrin and GABA_A_ receptor. Additionally, NLGN2 provides trans-synaptic adhesion through the binding of presynaptic neurexins. In the cortex or hippocampus, NLGN2, similarly to neuroligin-1, was found in many proteolytic forms, e.g., as a truncated membrane protein or a soluble extracellular domain [181]. Interestingly, stimulation of NMDA receptors in cortico-hippocampal synaptoneurosomes induced proteolytic cleavage of NLGN2 that decreased its membrane level [63]. Furthermore, this process was blocked by MMP inhibition. Because C-terminal intracellular NLGN2 domain recruits gephyrin, collybistin, and GABA_A_ receptors to postsynaptic density, MMP-dependent proteolytic cleavage of NLGN2 may cause receptor dispersal from the synapse. This, in turn, may result in synapse remodeling, change in subunit composition of synaptic receptors, and long-term plasticity. However, identifying the member of the MMP family responsible for NMDAR-dependent NLGN2 cleavage upon heterosynaptic GABAergic plasticity requires further studies.

Metalloproteinase 1 (MMP1) is expressed mainly by astrocytes; it is responsible for the activation of protease-activated receptor 1 (PAR1) [182]. The involvement of PAR1 in GABAergic plasticity was described in cultured hippocampal neurons [183], in which it induced suppression of synaptic inhibition through the stimulation of endocannabinoid synthesis. Furthermore, studies performed in mice globally overexpressing MMP1 showed that MMP1-induced activation of PAR1 gives rise to increased frequency of inhibitory synaptic currents in the striatum [184]. In addition, activation of astrocytic PAR1 increased the activity of the calcium-activated anion channel Bestrophin-1, which is responsible for releasing GABA from astrocytes, thereby regulating GABAergic tonic inhibition in neurons [185]. Because GABAergic tonic current shows potent plasticity that often correlates with learning [186], it would be interesting to define the role of MMPs in controlling this phenomenon. In general, tonic inhibition is a potent regulator of neuronal excitability and thereby, indirectly, can control stimuli, which induce synaptic plasticity [187,188]. Interestingly, it was shown in the hippocampus that, among other proteases, MMP3 can also activate PAR1 in vitro [118]. Together, these findings underscore the importance of PAR-1 in GABAergic phasic and tonic inhibition, but the identity of the whole repertoire of proteases activating PAR-1 still awaits a detailed investigation.

## 4. Concluding Remarks and Future Directions

In recent years, significant progress has been made in our understanding of MMP9 and MMP3 roles in brain functioning, particularly in different forms of long-term synaptic plasticity and distinct types of memory. Based on these results, there is a growing understanding of the role of MMPs in many brain pathologies such as, among others, Alzheimer’s disease [10], schizophrenia [189], epilepsy [190,191] and addiction development [5]. Despite the significant progress, numerous questions remain to be addressed. For example, how do different synaptic extracellular proteases such as MMP3, MMP9, tPA, neuropsin, and other metzincins directly or indirectly influence each other? What is the precise spatial and temporal profile of MMPs activity in the vicinity of synapses? Which matricryptins are produced by proteolytic activity of synaptic or perisynaptic proteases, and what are their precise functions? How does the activity of endogenous perisynaptic proteases relate to the synaptic and learning-related effects of exogenous chondroitinase or hyaluronidase?

Although MMPs have been implicated in synaptic plasticity at excitatory synapses for many years, it is only recently that it has been appreciated that metalloproteinases also play significant roles in inhibitory synapses. Recent research has demonstrated that MMP3 activity is necessary for heterosynaptic GABAergic plasticity by controlling the lateral membrane diffusion and synaptic trapping of GABA_A_ receptors [19]. This unique role of MMP3 underscores the intricate relationship between extracellular proteolysis and activity-dependent changes in the pool of synaptic receptors, which is critical for almost all forms of postsynaptic plasticity. Interestingly, the impaired GABAergic plasticity and l-type-dependent excitatory LTP in MMP3-deficient mice are accompanied by an enhanced classical conditioning and spatial learning. This intriguing phenotype offers a unique opportunity to study the role of inhibitory plasticity during the process of learning. Furthermore, we could speculate that enhanced learning in MMP3-deficient mice might be related to the engram size, which may depend on the extent of iLTP [147,192]. Thus, impaired iLTP in MMP3-null mice could abolish the restriction imposed by strengthened synaptic inhibition and increase the number of neurons recruited into the engram, resulting in memory enhancement and faster learning observed in MMP3-deficient mice. Future studies should shed more light on the relationship between extracellular proteolysis, synaptic plasticity, and engram size. Finally, our growing understanding of mechanisms whereby specific MMPs shape memory and learning together with the development of pharmacological tools able to manipulate the activity of proteases (e.g., with small molecule inhibitors, increasing the specificity of available MMP9 or MMP3 inhibitors) is expected to open new avenues in therapeutic strategies aiming at memory enhancement in neurological patients or controlling various types of addiction.

## Data Availability

Not applicable.
